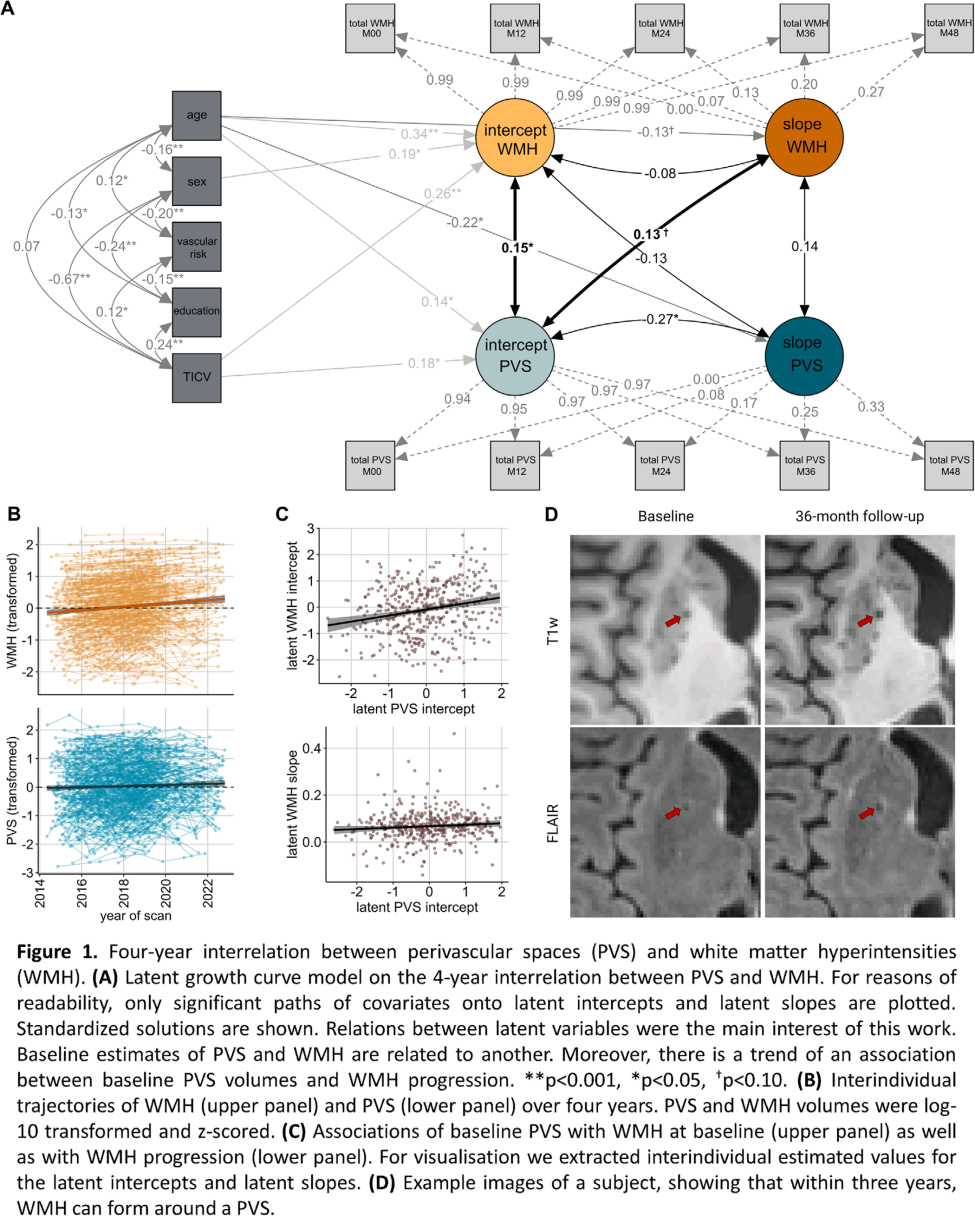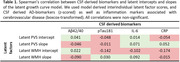# Interrelationship between perivascular spaces and white matter hyperintensities: A latent growth curve analysis

**DOI:** 10.1002/alz.095224

**Published:** 2025-01-09

**Authors:** Inga Menze, Jose Bernal Moyano, Pinar Kaya, Cagla Aki, Malte Pfister, Jonas Geisendörfer, Renat Yakupov, Roberto Duarte, Maria del C. Valdes Hernandez, Michael T. Heneka, Frederic Brosseron, Matthias Schmid, Wenzel Glanz, Enise I Incesoy, Michaela Butryn, Ayda Rostamzadeh, Dix U. Meiberth, Oliver Peters, Lukas Preis, Dominik Lammerding, Daria Gref, Josef Priller, Eike Jakob Spruth, Slawek Altenstein, Andrea Lohse, Stefan Hetzer, Anja Schneider, Klaus Fliessbach, Okka Kimmich, Ina R Vogt, Jens Wiltfang, Claudia Bartels, Björn H. Schott, Niels Hansen, Peter Dechent, Katharina Buerger, Daniel Janowitz, Robert Perneczky, Boris‐Stephan Rauchmann, Stefan Teipel, Ingo Kilimann, Doreen Goerss, Christoph Laske, Matthias H. J. Munk, Carolin Sanzenbacher, Petra Hinderer, Klaus Scheffler, Annika Spottke, Nina Roy‐Kluth, Falk Lüsebrink, Katja Neumann, Joanna M Wardlaw, Frank Jessen, Stefanie Schreiber, Emrah Düzel, Gabriel Ziegler

**Affiliations:** ^1^ German Center for Neurodegenerative Diseases (DZNE), Magdeburg Germany; ^2^ Institute of Cognitive Neurology and Dementia Research (IKND), Otto‐von‐Guericke University, Magdeburg Germany; ^3^ Centre for Clinical Brain Sciences, The University of Edinburgh, Edinburgh, Scotland United Kingdom; ^4^ Department of Neurology, Otto‐von‐Guericke University, Magdeburg Germany; ^5^ Luxembourg Centre for Systems Biomedicine (LCSB), University of Luxembourg, Luxembourg Luxembourg; ^6^ German Center for Neurodegenerative Diseases (DZNE), Bonn Germany; ^7^ Institute of Medical Biometry, Informatics and Epidemiology, University Hospital Bonn, Bonn Germany; ^8^ Department of Psychiatry and Psychotherapy, Otto‐von‐Guericke University, Magdeburg Germany; ^9^ Department of Psychiatry and Psychotherapy, Medical Faculty, University of Cologne, Cologne Germany; ^10^ Charité – Universitätsmedizin Berlin, corporate member of Freie Universität Berlin and Humboldt‐Universität zu Berlin – Institute of Psychiatry and Psychotherapy, Berlin Germany; ^11^ German Center for Neurodegenerative Diseases (DZNE), Berlin Germany; ^12^ School of Medicine, Technical University of Munich; Department of Psychiatry and Psychotherapy, Munich Germany; ^13^ University of Edinburgh and UK DRI, Edinburgh United Kingdom; ^14^ Department of Psychiatry and Psychotherapy, Charité, Berlin Germany; ^15^ Berlin Center for Advanced Neuroimaging, Charité – Universitätsmedizin Berlin, Berlin Germany; ^16^ Department of Neurodegenerative Diseases and Geriatric Psychiatry, University of Bonn Medical Center, Bonn Germany; ^17^ Department of Psychiatry and Psychotherapy, University Medical Center, University of Goettingen, Goettingen Germany; ^18^ German Center for Neurodegenerative Diseases (DZNE), Goettingen Germany; ^19^ Neurosciences and Signaling Group, Institute of Biomedicine (iBiMED), Department of Medical Sciences, University of Aveiro, Aveiro Portugal; ^20^ Leibniz Institute for Neurobiology, Magdeburg Germany; ^21^ 21MR‐Research in Neurosciences, Department of Cognitive Neurology, Georg‐August‐University Goettingen, Goettingen Germany; ^22^ Institute for Stroke and Dementia Research (ISD), University Hospital, LMU, Munich Germany; ^23^ German Center for Neurodegenerative Diseases (DZNE), Munich Germany; ^24^ Imperial College London, London United Kingdom; ^25^ Munich Cluster for Systems Neurology (SyNergy), Munich Germany; ^26^ Department of Psychiatry and Psychotherapy, University Hospital, LMU Munich, Munich Germany; ^27^ Sheffield Institute for Translational Neuroscience, University of Sheffield, Sheffield United Kingdom; ^28^ Department of Neuroradiology, LMU University Hospital, Munich, Germany, Munich Germany; ^29^ Department of Psychosomatic Medicine, Rostock University Medical Center, Rostock Germany; ^30^ German Center for Neurodegenerative Diseases (DZNE), Rostock Germany; ^31^ German Center for Neurodegenerative Diseases (DZNE), Tuebingen Germany; ^32^ Section for Dementia Research, Hertie Institute for Clinical Brain Research and Department of Psychiatry and Psychotherapy, University of Tuebingen, Tuebingen Germany; ^33^ Department of Psychiatry and Psychotherapy, University of Tuebingen, Tuebingen Germany; ^34^ Department of Biomedical Magnetic Resonance, Tuebingen Germany; ^35^ Department of Neurology, University of Bonn, Bonn Germany; ^36^ Excellence Cluster on Cellular Stress Responses in Aging‐Associated Diseases (CECAD), University of Cologne, Cologne Germany

## Abstract

**Background:**

Inadequate glymphatic clearance through perivascular spaces (PVS) is hypothesized to contribute to the formation of white matter hyperintensities (WMH). However, longitudinal evidence for such a mechanistic link in aging remains limited. Using multivariate modelling, we investigated the interrelationship between PVS and WMH over time to elucidate potential cascades of early cerebrovascular alterations and tested whether AD‐biomarkers and inflammatory markers associated with vascular disease can explain individual variability in their occurrence and progression.

**Methods:**

We quantified PVS and WMH using T1w MPRAGE and T2w FLAIR imaging of 439 cognitively unimpaired participants from the DELCODE study (52.85% females; mean_age_ = 69.88±5.72), who underwent annual scans over a four‐year period and attended at least three visits (*n*_observations_ = 1790; mean_number of visits_ = 4.08±0.79). We employed latent growth curve modelling to assess reciprocal connections between PVS and WMH, focusing on their initial volumes (latent intercepts) and their rates of change over four years (latent slopes). We used log10‐transformed total PVS and WMH volumes, and controlled for age, sex, years of education, total cardiovascular risk score, and total intracranial volume. We then derived interindividual latent factor scores and tested their relation to CSF‐derived AD‐biomarkers (Aβ42/40, pTau181; available for *n* = 195; z‐scored) and inflammatory markers (CRP, IL‐6; available for *n* = 125; Box‐Cox‐transformed) via Spearman’s correlation (FDR‐corrected).

**Results:**

The model showed good model fit (*CFI* = 0.997; *RMSEA* = 0.021; *SRMR* = 0.017; **Fig. 1A**). WMH and PVS volumes increased over time (*intercept_WMH‐slope_* = 0.068, *SE* = 0.004, *Z* = 16.490, *p<*0.001; *intercept_PVS‐slope_* = 0.036, *SE* = 0.007, *Z* = 4.927, *p<*0.001; **Fig. 1B**). Participants with higher baseline PVS volumes not only had higher baseline WMH volumes (*covariance_PVS‐intercept&WMH‐intercept_* = 0.120, *SE* = 0.040, *Z* = 2.936, *p =* 0.003; **Fig. 1C**) but also tended to exhibit faster WMH volume increase over time (*covariance_PVS‐intercept&WMH‐slope_* = 0.007, *SE* = 0.004, *Z* = 1.796, *p =* 0.072; **Fig. 1C**). In this sample of cognitively unimpaired participants, biomarkers of AD and inflammation did neither relate to individual baseline differences nor progression rates (**Table 1**).

**Conclusion:**

Our findings are consistent with the notion that PVS dysfunction might contribute to and precede WMH progression (**Fig. 1D**). However, the individual variability requires further investigation to elucidate mechanisms driving PVS dysfunction in the first place. Unraveling the interrelationships and further factors contributing to cerebrovascular alterations will be crucial to understand pathological cascades in aging that could inform targeted treatment strategies.